# Partial abdominal evisceration and intestinal autotransplantation to resect a mesenteric carcinoid tumor

**DOI:** 10.1186/1477-7819-9-11

**Published:** 2011-01-31

**Authors:** William H Kitchens, Nahel Elias, Lawrence S Blaszkowsky, A Benedict Cosimi, Martin Hertl

**Affiliations:** 1 Department of Surgery, Massachusetts General Hospital, Boston, MA, USA; 2 Transplantation Unit, Department of Surgery, Massachusetts General Hospital, Boston, MA, USA; 3 Massachusetts General Hospital Cancer Center, Boston, MA, USA

## Abstract

**Background:**

Midgut carcinoids are neuroendocrine tumors that commonly metastasize to the intestinal mesentery, where they predispose to intestinal obstruction, ischemia and/or congestion. Because of their location, many mesenteric carcinoid tumors are deemed unresectable due to the risk of uncontrollable bleeding and prolonged intestinal ischemia.

**Case Presentation:**

We report the case of a 60-year-old male with a mesenteric carcinoid tumor obstructing his superior mesenteric vein, resulting in intestinal varices and severe recurrent GI bleeds. While his tumor was thought to be unresectable by conventional techniques, it was successfully resected using intestinal autotransplantation to safely gain access to the tumor. This case is the first described application of this technique to carcinoid tumors.

**Conclusions:**

Intestinal autotransplantation can be utilized to safely resect mesenteric carcinoid tumors from patients who were not previously thought to be surgical candidates. We review the literature concerning both carcinoid metastases to the intestinal mesentery and the use of intestinal autotransplantation to treat lesions involving the mesenteric root.

## Background

Carcinoid tumors are the most common neuroendocrine neoplasm, typically arising in the respiratory and midgut gastrointestinal tracts [1,2]. While midgut carcinoid tumors are usually slow growing, they are often associated with severe complications from early metastasis to the liver and the small bowel mesentery [3]. These mesenteric carcinoid tumors release serotonin and other growth factors which induce a desmoplastic reaction causing diffuse mesenteric fibrosis and encasement of critical mesenteric vasculature, which in turn predisposes to intestinal obstruction, hypoperfusion and/or congestion [4]. Several groups have published surgical strategies for debulking mesenteric carcinoid disease [5-7]. However, complete encasement of the mesenteric vasculature has traditionally been considered an absolute contraindication to surgery given the risk of uncontrollable bleeding or inducing prolonged intestinal ischemia [3]. Here we describe a novel technique to resect extensive mesenteric root carcinoid metastases using partial abdominal evisceration and intestinal autotransplantation.

## Case presentation

**Figure 1:**
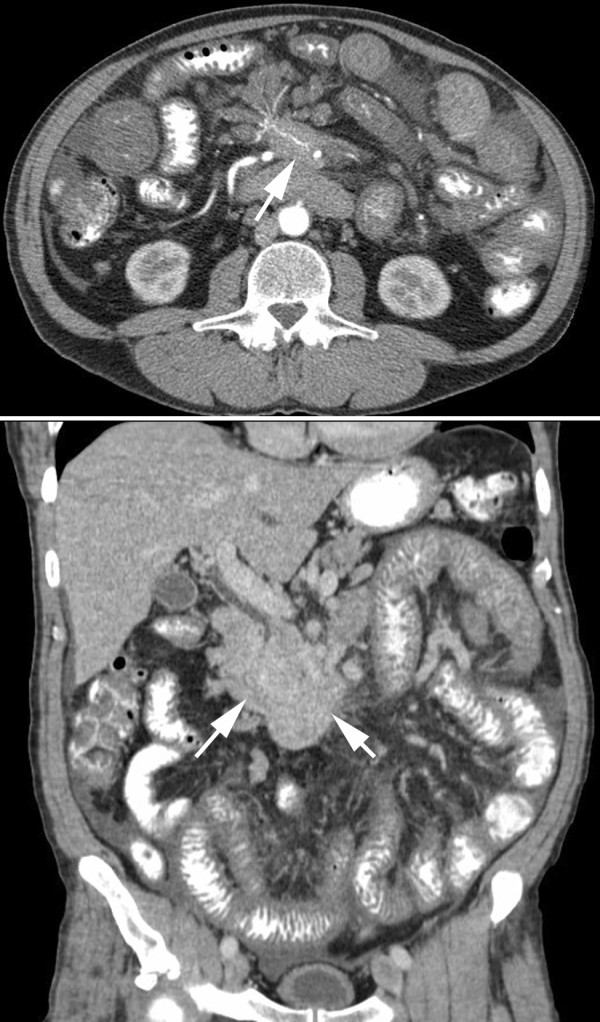
**Preoperative abdominal CT scan demonstrating large mesenteric soft tissue mass (white arrows) encasing the mesenteric vasculature with evident bowel wall and mesenteric edema, along with trace ascites fluid**.

**Figure 2:**
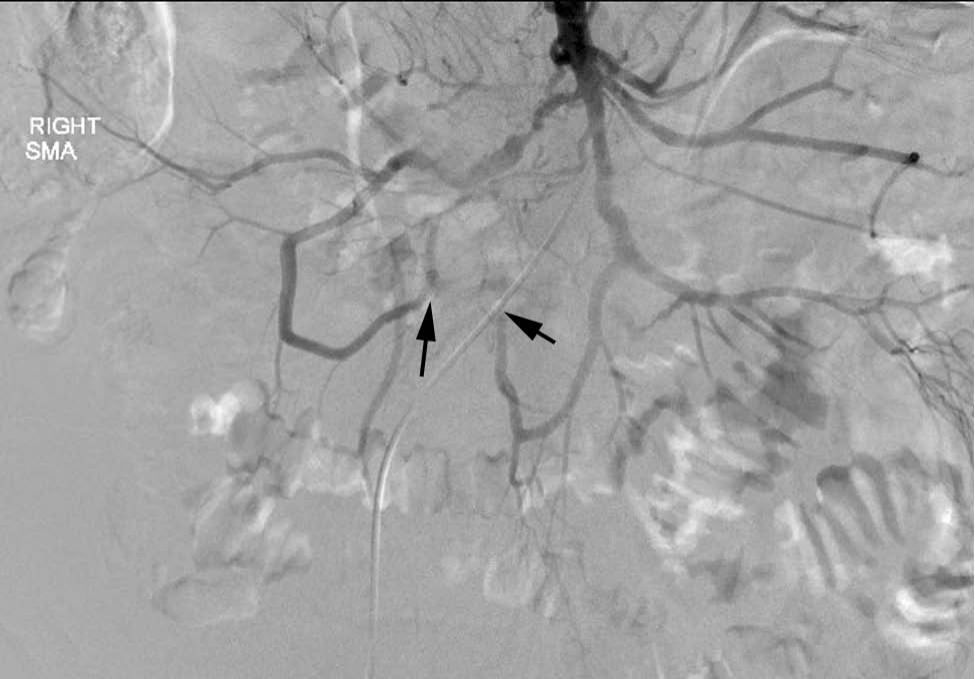
**Pre-operative mesenteric angiogram**. Whereas the celiac and inferior mesenteric arteries were patent, cannulation of the superior mesenteric artery shown here reveals severe stenosis or occlusion of multiple SMA branches (black arrows) from extrinsic compression by the carcinoid tumor. Flow to the ileocolic artery is occluded proximally and reconstituted distally from jejunoileal collaterals

A 60-year-old man presented to our unit in January 2008 with a mesenteric carcinoid tumor compressing the superior mesenteric vein, resulting in recurrent episodes of GI bleeding from mesenteric varices. His relevant medical history began in 2005, when he underwent a work-up at another institution for chronic diarrhea at that time. He complained of up to 15 watery bowel movements a day for the past several years. He underwent an unrevealing esophagogastroduodenoscopy (EGD) and colonoscopy, but an abdominal CT scan showed a 4.6 × 2.5 cm mesenteric soft tissue mass encasing the superior mesenteric vein and extending along the mesenteric root to the base of the pancreas (Figure 1). Multiple mesenteric varices were noted on this original CT scan, and several small bowel loops appeared thickened with probable venous congestion. Both 24-hour urine 5-HIAA (14.7 mg, normal 2-6 mg) and chromogranin A levels (76.7 ng/mL, normal < 36.4 ng/mL) were elevated, consistent with a diagnosis of mesenteric carcinoid tumor. His tumor was deemed inoperable after the outside institution's evaluation, and he was referred to a medical oncologist at our hospital. Additional studies then included an EGD with endoscopic ultrasound (EUS) and a fine needle-aspiration biopsy of the mass. These showed a 3.5 cm mass encasing the SMV, and the biopsy confirmed that he had a chromogranin-positive, synaptophysin-positive, keratin AE1/AE3-positive, NSE-negative well-differentiated carcinoid tumor without evidence of atypia (no mitoses or necrosis, ENETS grade G1). Mesenteric angiography revealed severe stenosis or occlusion of multiple SMA branches such as the ileocolic artery, which was reconstituted distally by jejunoileal collaterals (Figure 2). Staging chest/abdomen/pelvis CT scans showed no evidence of metastatic disease. He was offered participation in a clinical trial but declined, choosing instead to receive standard therapy with octreotide long-acting release (LAR).

His disease remained fairly stable for almost two years on octreotide, with only slight progressive enlargement of his mesenteric mass on interval abdominal CT scans. Starting in August 2007, however, he required multiple hospital admissions for GI bleeding. Colonoscopy was unremarkable, and an EGD showed thickened folds in the second portion of the duodenum. A video-capsule endoscopy revealed diffuse congested mucosa throughout the jejunum, with a few localized erosions. A push enteroscopy was performed, but his duodenal and jejunal varices were not amenable to intervention. By January 2008, he was requiring 2-4 units of red blood cell transfusions a week due to continued GI bleeding; a palliative care consult was obtained and preparations were made to proceed with home hospice. At this point he was referred for a second surgical opinion. Given his otherwise state of good health and his desire for aggressive treatment of his carcinoid tumor, it was determined that we should attempt a palliative resection including partial abdominal evisceration for resection of his mesenteric carcinoid tumor and reconstruction via intestinal autotransplantation.

### Operative technique

**Figure 3:**
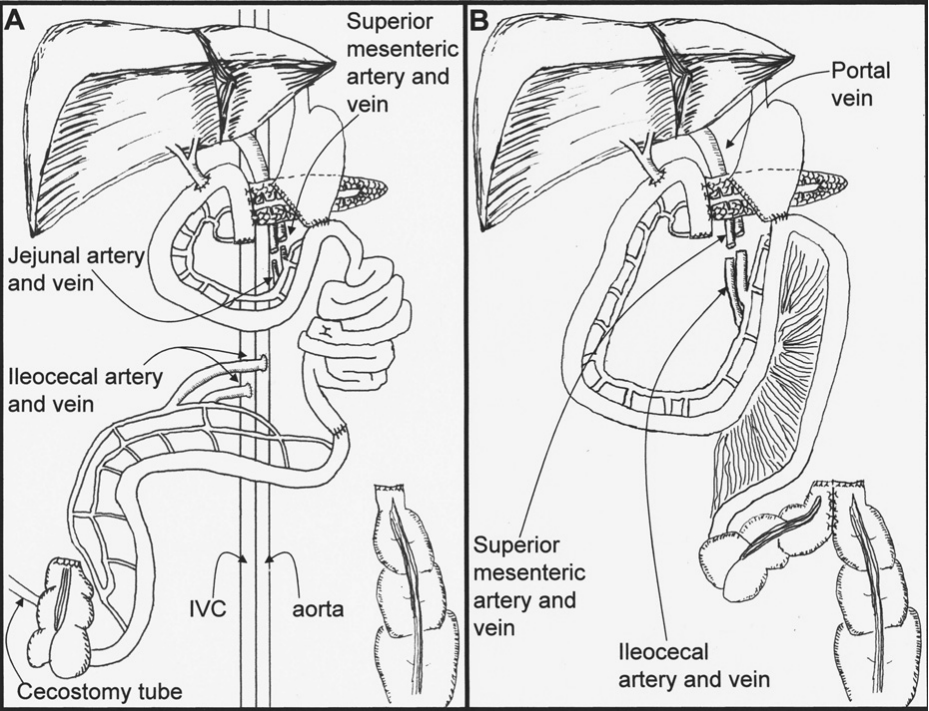
***A*. Initial postoperative anatomy**. Both ileocecal and ileojejunal segments of bowel were removed to permit access and resection of the mesenteric carcinoid tumor. The ileojejunal autograft was revascularized by anastomosing the jejunal artery and vein to the residual stumps of the SMA and SMV. The ileocecal segment was revascularized by anastomosing the ileocolic vessels to the abdominal aorta and IVC. The GI tract was reconstructed with a pancreaticojejunostomy, hepaticojejunostomy, gastrojejunostomy and ileoileostomy. *B*. Final postoperative anatomy. Due to SMA thrombosis, the jejunoileal bowel autograft was lost. The ileocolic segment was explanted and revascularized using the SMA and SMV stumps. GI tract continuity was restored with a pancreaticoileostomy, hepaticoileostomy, gastroileostomy and cecocolostomy

Laparotomy was performed through a bilateral subcostal incision. The carcinoid tumor was confirmed to involve the root of the small bowel mesentery, extending down to the aorta and vena cava, accompanied by lymphadenopathy of the para-aortic nodes. There was no evidence of hepatic metastasis, but the duodenum and head of the pancreas were involved. The mesentery of the ileum and jejunum was investigated to find suitable vessels for anastomosis. The tumor extended past the confluence of the ileocolic artery with the first jejunal artery branch, therefore requiring that the intestine be autotransplanted in two separate segments. The distal ileocolic artery was deemed suitable to support the cecum and terminal ileum, and the jejunal artery was selected to support a large segment of jejunum and proximal ileum. The Kocher maneuver was performed, followed by a radical lymphadenectomy along the vena cava and aorta. A pre-pyloric division of the stomach was made, and the jejunum was divided just distal to the ligament of Treitz. The common hepatic bile duct was divided distally and the pancreaticoduodenectomy was completed by dividing the neck of the pancreas. At this time, both the ileocecal and ileojejunal segments of bowel were removed and transported to the back table, where they were each flushed with 2 L of UW preservation solution and kept on ice. The resection continued by transecting the superior mesenteric artery, leaving a stump of about 2 cm behind. The portal vein was dissected free and then the superior mesenteric vein was divided 2 cm proximal to its confluence with the splenic vein. The descending colon was stapled off just distal to the splenic flexure. The resected specimen included the mesenteric root, pancreatic head, duodenum and ascending/transverse colon. The ileojejunal segment of intestine was then brought back to the field and was revascularized via the retained superior mesenteric artery stump and the transsected distal superior mesenteric vein. The bowel reperfused well. Next, the infrarenal vena cava and the aorta below the take-off of the inferior mesenteric artery were utilized to complete end-to-side vascular anastomosis for the ileocolic segment of bowel. This bowel segment also reperfused well. GI tract continuity was completed using a pancreaticojejunostomy, gastrojejunostomy and choledochojejunostomy and an end-to-end ileoileostomy to connect the two segments of autotransplanted bowel. The descending colon was left stapled off, and a Bardex cecostomy tube was brought out through the skin to temporarily drain the cecum (Figure 3A). The abdomen was irrigated and closed in layers.

### Post-operative course

Final pathology revealed well-differentiated multifocal carcinoid of the small bowel with metastases to the mesentery (where a 6.5 cm mass was identified) and mesenteric lymph nodes (9 of 18 nodes positive). All resection margins were negative for malignancy. By TNM staging (as defined by ENETS), his tumor was stage IIIB. The patient had a complicated post-operative course. A second-look laparotomy performed on postoperative day 1 revealed all of the bowel to be viable. The patient was brought back to the operating room on postoperative day 4 with plans to connect the cecum and descending colon. At this laparotomy, the ileojejunal segment of bowel looked dusky, albeit not frankly necrotic. The ileocolic segment remained pink and well-perfused. A near-occlusive clot was found at the superior mesenteric artery anastomosis. Fogarty thrombectomy restored good flow to that bowel segment. Continued acidosis and hemodynamic instability required re-exploration the next day, at which point a large thrombus was again palpated in the SMA, and the ileojejunal segment of bowel was found to be nonviable. This segment of transplanted bowel was therefore resected, requiring take-down of the pancreaticojejunostomy, choledochojejunostomy and gastrojejunostomy. Reconstruction could not be performed with the ileocecal segment of bowel in its current location, so this segment was explanted, flushed with ice-cold lactated Ringer solution containing mannitol and then kept on ice. The venotomy and arteriotomy on the vena cava and abdominal aorta were patched with pieces of ileal vein and artery, respectively. The ileocecal segment (including ~70 cm of ileum) was then revascularized via the superior mesenteric artery and vein. The bowel was pink immediately upon reperfusion. A pancreaticoileostomy, gastroileostomy and choledochoileostomy were then performed in the standard fashion. Reconstruction was completed by anastomosing the cecum to the descending colon (Figure 3B). Postoperatively, he was started on a heparin drip to prevent further thrombosis and he was successfully extubated the next day.

His subsequent hospital course was remarkable for periodic febrile episodes requiring first IR drain placement into a pelvic fluid collection (which grew *Candida albicans*) and a final laparotomy for abdominal washout with saline containing amphotericin B and gentamicin. He did have a small leak at his pancreaticoileostomy that was managed conservatively with NPO status and initiation of TPN. He was discharged on TPN on POD 72, tolerating some tube feeds and enteral intake.

### Outcome

Over the ensuing months the patient required several short hospitalizations for central venous line infections related to his TPN, in addition to endocarditis. He was ultimately weaned off parenteral nutrition and his weight has stabilized. He had recovered well enough to undergo an aortic valve replacement in December 2009. Currently, 2.5 years after his carcinoid resection, he is completely independent of parenteral nutrition or tube feeds. He continues to have mild diarrhea, well-controlled with cholestyramine. Surveillance abdominal CT scans reveal no evidence of carcinoid recurrence or metastasis, and his chromogranin A in March 2010 was within normal limits (93 ng/ml, reference < 225 ng/ml).

## Discussion

Of the gastrointestinal carcinoids, those arising from the midgut (*e.g. *small intestine and appendix) are by far the most common, with tumors distal to the jejunum representing 96% of carcinoids located in the gastrointestinal tract [8]. These midgut carcinoids secrete serotonin and are responsible for the carcinoid syndrome, the intractable flushing and diarrhea associated with metastasis to the liver. In addition to hepatic metastasis, midgut carcinoids also commonly metastasize to the small bowel mesentery [3]. Indeed, radiographic studies indicate mesenteric involvement in 40-80% of patients with abdominal carcinoid tumors [9-11].

Carcinoid metastases to the mesentery often grow far larger than the submucosal primary tumors in the small bowel wall, and they are responsible for much of the morbidity and mortality of gastrointestinal carcinoid tumor that is not attributable to carcinoid syndrome itself. Many patients with mesenteric carcinoid present with small bowel obstructive symptoms due to tethering and kinking of the small bowel to the rigid mesentery. In several surgical case series of patients with midgut carcinoids, 62-67% required laparotomy for either intestinal obstruction or abdominal pain, and of these patients, 67-79% had evidence of extensive mesenteric fibrosis upon surgical exploration [12,13]. In other cases, the mesenteric vasculature (*e.g. *superior mesenteric artery and vein) can become completely encased in tumor, causing regional portal hypertension and arterial insufficiency [14-16]. The encasement of the visceral vasculature may manifest as episodes of post-prandial acute abdominal pain or as GI bleeds (noted in 5% of midgut carcinoid patients in one case series) [3,13].

Although somatostatin analogs such as octreotide remain the mainstay of carcinoid therapy, surgical resection has emerged as a vital treatment in disease management. For metastatic disease, palliative cytoreductive surgery has been employed by some groups to specifically address mesenteric carcinoids. While some symptomatic improvement is often reported, long-term outcomes are compromised by recurrent disease [5,17,18]. Unfortunately, large mesenteric carcinoids have often been considered unresectable due to their position abutting critical vascular structures in the abdomen.

Recently, however, several groups have published reports outlining the use of intestinal autotransplantation to safely gain access and resect tumors that encase the vasculature of the mesenteric root. Utilizing techniques from intestinal allotransplantation, the patient's small intestine is harvested *en bloc *and maintained in cold preservative fluid to limit warm ischemia injury. The mesenteric mass is then fully excised, and the intestine is autotransplanted back into the patient. This strategy was first employed by David Lai and colleagues to treat a nonfunctioning islet cell carcinoma [19]. That patient also underwent a total pancreatectomy, gastrectomy, splenectomy and hepatic revascularization (as his proximal hepatic artery was also resected). Vascular anastomoses were performed between the hepatic artery and aorta, distal superior mesenteric artery and aorta, and between the distal superior mesenteric vein and cephalad portal vein.

A similar approach was employed by Tzakis and colleagues to treat four patients with lesions involving the root of the mesentery. The indications for resection in these patients were pancreatic head fibroma, vascular malformation at the mesenteric root, desmoid tumor of the pancreatic tail, and locally advanced pancreatic adenocarcinoma [20,21]. Whereas we employed *in situ *resection of the carcinoid tumor, the patients in Tzakis' case series had *ex vivo *resection of their lesions on ice on the back table after their organs had been removed *en bloc*. The mesenteric lesions of his series did not apparently extend into the distal branches of the SMA, obviating the need to autotransplant multiple segments of intestine as in the case we present here. All of his patients had good outcomes except for the patient treated for pancreatic cancer, who died of hepatic metastases 7 months postoperatively. Two additional case reports of intestinal autotransplantation to treat locally-advanced pancreatic cancer extending into the mesenteric root were also complicated by rapid recurrence of hepatic or peritoneal metastases, and early death after the procedure [22,23]. These reports cast doubt on whether this aggressive surgical strategy is warranted for cases of pancreatic cancer.

## Conclusion

Here we present the first description of partial abdominal evisceration and intestinal autotransplantation used to treat a mesenteric carcinoid tumor. This method may prove particularly useful for midgut carcinoid tumors, as this malignancy frequently extends into the mesenteric root, with enormous clinical consequences. While stenting of the superior mesenteric vein was recently described to palliate the symptoms of intestinal congestion that often accompany mesenteric spread of midgut carcinoids [24], intestinal autotransplantation permits tumor resection with possible curative results. In conjunction with earlier studies, this report demonstrates that intestinal autotransplantation is a surgical strategy that can be used to successfully treat a variety of lesions involving the mesenteric root, including many that were previously thought to be unresectable.

## List of abbreviations

EGD: esophagogastroduodenoscopy; EUS: endoscopic ultrasound; SMA: superior mesenteric artery; SMV: superior mesenteric vein; TPN: total parenteral nutrition

## Competing interests

The authors declare that they have no competing interests.

## Authors' contributions

WHK prepared the manuscript and all illustrations contained therein. MH was the primary surgeon and helped to develop the surgical technique. ABC and NE were co-surgeons involved in the development of the surgical technique, and ABC also provided critical help revising the manuscript. LSB was the medical oncologist of the patient and contributed to his preoperative and postoperative care. All authors read and approved the final manuscript.

## Consent

Written consent was obtained from the patient for publication of this case report and the accompanying images. A copy of the written consent is available for review by the Editor-in-Chief of this journal.
